# Anatomy, Morphology and Function of the Tensor of Vastus Intermedius: A Systematic Review

**DOI:** 10.3390/jfmk6030077

**Published:** 2021-09-16

**Authors:** Chrysostomos Sahinis, Eleftherios Kellis

**Affiliations:** Laboratory of Neuromechanics, Department of Physical Education and Sport Sciences at Serres, Aristotle University of Thessaloniki, Agios Ioannis, 62100 Serres, Greece; ekellis@phed-sr.auth.gr

**Keywords:** systematic review, evidence-based anatomy, tensor of vastus intermedius, quadriceps, vastus lateralis, vastus intermedius

## Abstract

The tensor of vastus intermedius is a newly discovered muscle that is located at the anterior compartment of the thigh. The aim of the present study is to report, assess and synthetize the existing evidence on the anatomy, variation and morphological characteristics of the TVI as well as to examine its clinical importance. A systematic review was performed evaluating both anatomical and medical imaging studies which provided information about TVI anatomy, prevalence, variations and morphological characteristics. The search strategy was conducted in major electronic databases. Two reviewers worked independently to screen all possible references via a title/abstract examination. Methodological quality was examined with the Anatomical Quality Assurance checklist. A total of 295 cadaveric knees were included in the nine studies where in 244 (82.7%) cases the TVI was identified. Based on this evidence, it appears that the TVI is located between the vastus lateralis and vastus intermedius. The muscle belly is located proximally, and it is combined with a broad and flat aponeurosis before forming a tendinous structure that is attached at the medial aspect of the patella. The TVI presented some morphological variations and complex muscle architecture that varied along its length. There is insufficient good quality evidence as more than half of the included studies were ranked as having a “High” risk of bias with various methodological issues. Higher quality studies are recommended to evaluate the TVI morphology to better understand its functional and clinical importance.

## 1. Introduction

Anatomical variations are a common phenomenon in the human body and even though some have been extensively described in the literature, many are still unexplored. The appearance of accessory muscles, abnormal tendon course, or anomalous origin or insertion are frequently reported in lower limbs [1,2]. In particular, supplementary muscles are anatomic variants representing additional distinct muscles that are encountered between normal complement muscles [1,2]. Although they are generally asymptomatic and identified as incidental findings, sometimes they are considered a potential source of pain and clinical symptoms [3,4].

The quadriceps femoris (QF) is considered a dominant extensor of the knee and a flexor of the hip joint. The QF is composed structurally of four separate muscles, the vastus lateralis (VL), the vastus intermedius (VI), the vastus medialis (VM) and the rectus femoris (RF). In particular, the RF takes its origin from the anterior inferior iliac spine, the VL from the greater trochanter, the VM from the linear aspera, while the VI from the anterior and lateral shaft of the femur. Distally, all tendons unite to form a single tendon that attaches to the base of the patella. This common tendon was reported as a tri-laminar arrangement that is composed by RF superficially, vastus VL and VM in the intermediate and VI in the deepest layer [5,6,7] while others have failed to observe this structure reporting a more complex organization [8]. This morphological variability has also been described for QF musculature [9,10].

The complex of the QF is still under examination as it is uncertain whether the QF can be regarded as a group with four individual muscle heads [10,11]. Past literature noticed that it is possible that VM and VL are separated in two discrete parts [9]. Recent anatomical investigations documented the existence of a fifth head, the tensor of the vastus intermedius (TVI) which is located between the VL and the VI [10,12]. In particular, the TVI has been thoroughly described by Grob et al. [10] as a muscle which originates proximally from the anterolateral aspect of the greater trochanter and distally to the quadriceps tendon and insert into the medial aspect of the patella [10,12]. However, other studies did not recognize the TVI in all limbs [11,13,14,15] and, hence, it remains unclear whether TVI is another independent head of this muscle group or it is simply a variant of the remainder quadricep muscles.

Even when the TVI is identified, the reports on its exact morphology are not consistent. That is, the origins and the morphology of the TVI belly and tendons vary between individuals and between studies [10,11,12,16]. Hence, clear identification of the TVI as an independent muscle becomes more difficult. In turn the examination of the role of this muscle-tendon structure for the whole quadriceps femoris function becomes unclear.

In the past, identification of variations in anatomy was usually limited to unexpected findings at surgery or during the process of anatomical dissection [1]. Advancements in ultrasound (US) and magnetic resonance imaging (MRI) enabled us to recognize accessory anatomical structures [2,7,17,18,19]. Τhe appearance of the TVI has been evaluated using such diagnostic imaging techniques with high reliability [17,18,20,21] and this provides the opportunity to examine the shape and role of this function, if any, in vivo [18,20,22].

A thorough and complete understanding of the architecture and anatomy of the knee extensor mechanism is of paramount importance in both clinical and research practice. For instance, the quadriceps tendon, where TVI combined, has a pivotal role in many orthopedic procedures involving harvesting as a tendon graft [20] or tendon injury [21]. Furthermore, the presence of another muscle-tendon unit which potentially has an independent innervation may have implications for the whole quadriceps muscle function, which is currently unknown. While a recent review attempted to summarize mainly the anatomical studies regarding TVI musculature [22], there have been several new studies on TVI architecture and morphology which have not been taken into consideration [11,16,18,20] while the risk of bias assessment of the included studies was not as evident. Being a newly identified muscle-tendon entity, the amount of evidence is still scarce even though new evidence on TVI presence as part of the quadriceps muscle group is growing. Studies have also highlighted that a detailed description of TVI architecture is still lacking which is probably due to the presence of several variations of TVI [11,18]. Hence, updating the previously presented information and providing detailed examination not only of TVI anatomy but as well as its architecture [22] and its variants will assist in appreciation of the role of TVI for quadriceps function. Therefore, the aim of this review was to examine whether TVI is identifiable in all limbs, secondly, to examine whether this muscle has a consistent morphology in all individuals, and, finally, to summarize findings regarding the function of the TVI. This will allow the development of a more clinically useful and relevant classification of TVI musculature, permitting better evaluation of treatment plans and improvement of surgical performance in the anterior compartment of the thigh.

## 2. Materials and Methods

### 2.1. Criteria for Study Selection

Each study was independently evaluated by two authors (C.S. and E.K.). Studies were included in this systematic review if they (a) provided complete data on the prevalence of vastus intermedius (b) reported the anatomy and the morphological characteristics, and (c) provided information regarding the neurovascular supply of TVI. This systematic review included both cadaveric and imaging studies without age, gender or country of origin restriction. The following exclusion criteria were employed: (1) articles containing irrelevant or insufficient data; (2) studies not written in the English language; (3) reviews, case reports, letters to editors, thesis and conference abstracts; and (4) studies conducted on animals. Any differences in opinion concerning the eligibility of the studies were solved by consensus.

### 2.2. Search Strategy

This present review was conducted in accordance to the Preferred Reporting Items for Systematic Reviews and Meta-Analyses (PRISMA) guidelines. The following electronic databases were searched to identify all relevant articles up to 20 May 2021: PubMed and Scopus. An extensive literature search was carried out using specific syntax rules for each database and combining the following terms with adequate Boolean operators: “tensor of vastus intermedius”, “quadriceps anatomy”, “vastus intermedius”, “fifth head of quadriceps”, “quanticeps”, “extensor apparatus of the knee joint”, “quadriceps femoris variation”. To avoid the omission of any relevant publications these broad search terms were used. Search results were collected using a reference manager software (Mendeley, version: 1.19.2). Then, duplicates were removed and studies were systematically screened using titles and abstracts. All relevant studies were selected for the full-text assessment, based on predetermined exclusion and inclusion criteria. Furthermore, secondary searches were carried out by manual investigations on the reference lists of the included articles or by performing forward citation tracking through Google Scholar and Scopus to identify additional resources. Only studies from 2016 and after were included in this systematic review as at this year the TVI was formally identified and described by Grob et al. [10]. To mitigate the probability of study selection bias, two authors of the review performed the study selection independently. When both authors completed their investigations, the final lists of included and excluded studies were compared between them. Any discrepancies between studies were resolved through discussion and consensus. To facilitate literature analysis and synthesis, a PICOS framework (Patient/population: cadavers or healthy participants; Intervention: dissection or medical imaging assessment, Comparators: none; Outcomes: anatomy, prevalence, variations, morphological characteristics and function of the TVI, Studies: Original cadaveric or medical imaging reporting the above findings) was developed.

### 2.3. Risk of Bias Assessment

The AQUA tool was used by the reviewers to assess the quality and reliability of the included studies [23]. The AQUA tool probes for the potential risk of bias in five study domains, namely, (1) objectives and subject characteristics, (2) study design, (3) methodology characterization, (4) descriptive anatomy, and (5) reporting of results. The risk of bias within each domain is normally categorized as “Low”,” High”, or “Unclear”. If at least one of the signaling questions within each of the domains was answered with “No”, the reviewers arbitrated the domain to be of “High” risk of bias, while if all questions was answered with “Yes” then that domain is assigned to a “Low” risk of bias. For questions that could not be answered due to unreported or insufficient information, the risk of bias was defined as “High”.

### 2.4. Data Extraction

Relevant data from the included studies was extracted by 2 independent reviewers (C.S. and E.K.). In particular, the extracted data included: (a) author names and year of publication; (b) characteristics of the sample size, including their age, country of origin and gender; and (c) prevalence of TVI. Any disagreements during the extraction procedure were settled through discussion and consensus.

## 3. Results

### 3.1. Study Selection

**Figure 1:**
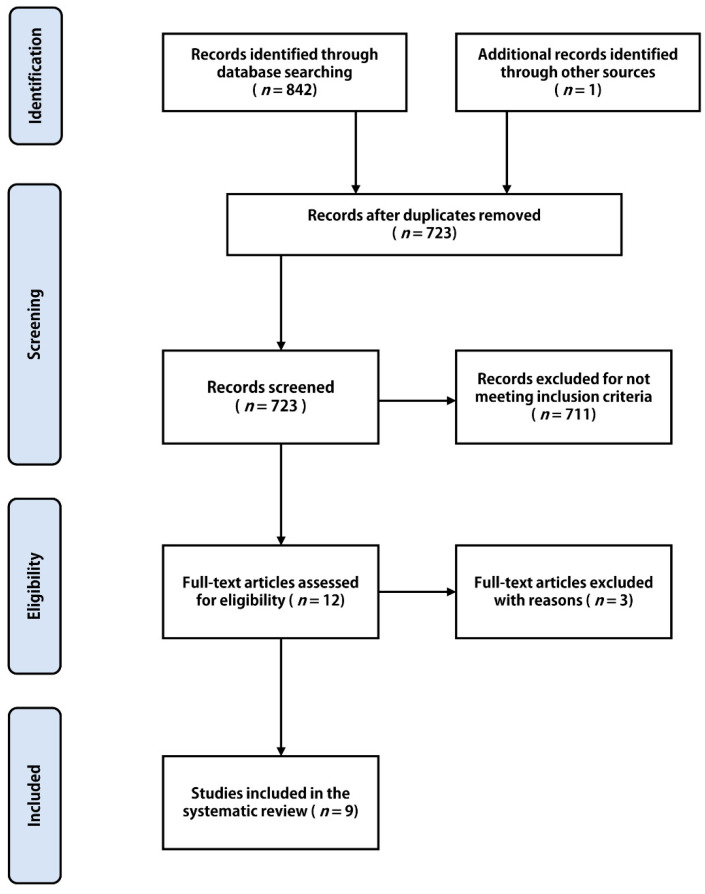
PRISMA flow chart of study identification and inclusion into the systematic review.

An adapted PRISMA flowchart summarizes the results of the study identification, screening, and eligibility evaluation (Figure 1). Through extensive searching of the online databases produced a total of 842 studies. Subsequently, duplicates were removed, giving a total of 723 studies eligible for title and abstract screening. After primary screening, a total of 12 articles were assessed using full-text, of which 3 articles were deemed ineligible. As a result, 9 articles (5 cadaveric studies and 4 imaging studies) met the criteria and were included in our analysis. The included studies are summarized in Table 1.

### 3.2. Criteria for Study Selection

**Figure 2:**
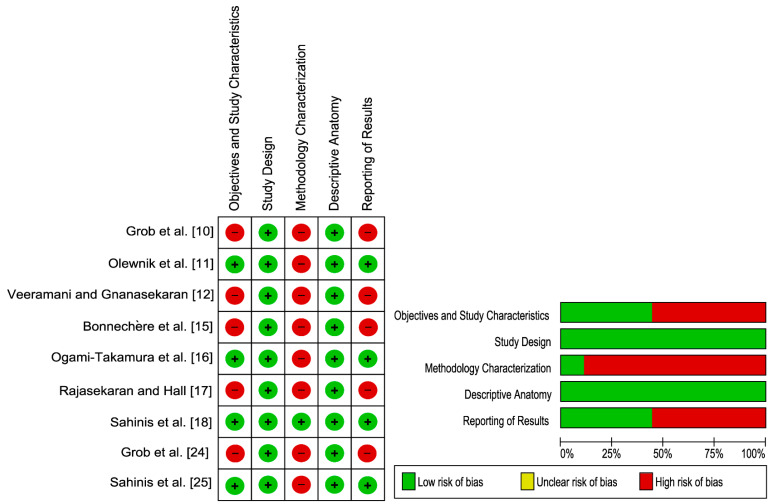
Quality assessment of the included studies [10,11,12,15,16,17,18,24,25] using AQUA tool.

The majority of the studies (*n* = 5) included in this systematic review, evaluated by the AQUA tool, revealed domain one (objective(s) and subject characteristics), domain three (methodology characterization) and domain five (reporting results) to be at “High” risk of bias (Figure 2). This was mainly due to missing demographic data of the study population especially in cadaveric studies, the lack of information regarding the experience of investigators as well as the paucity of information regarding the statistical analysis of the studies. Nevertheless, most of the studies had a “Low” risk of bias found in domain two (study design) and domain four (descriptive anatomy). The results of the risk of bias assessment of the individual studies can be found in Figure 2.

### 3.3. Characteristics of the Included Studies

**Figure 3:**
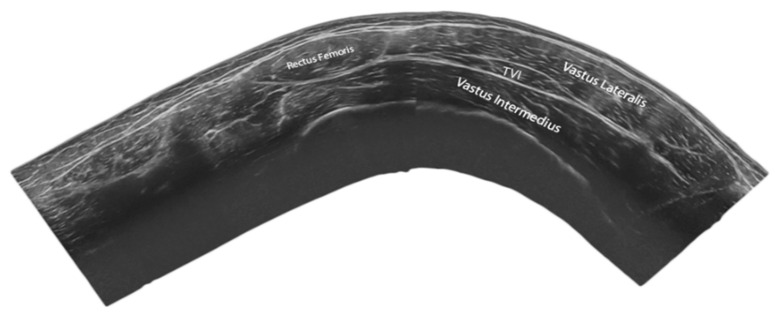
Representative extended field of the view ultrasound image of rectus femoris (RF), the vastus lateralis (VL), the vastus intermedius (VI) and the tensor of the vastus intermedius (TVI) obtained from the anterior region of the thigh obtained in our laboratory.

An overview of the study characteristics is shown in Table 1. Overall, 5 of the included studies were cadaveric [10,11,12,15,16] while the rest were imaging studies [17,18,20,21]. A total of 295 lower limbs were examined, of which 223 derived from cadaveric whilst only 72 derived from in vivo studies (Table 2). Assessment of TVI anatomy and morphology were performed through dissection and MRI evaluation in two studies [10,24], combined dissection and computed tomography in one study [16] whereas solely dissection was performed in three studies [11,12,15]. The in vivo examination of TVI were conducted using B-mode US in two studies [17,18] while one study used extended field-of-view US [25] (Figure 3).

### 3.4. Prevalence

The results of the identification rates of TVI between the studies were displayed in Table 2. Overall, the TVI was identified in 244 (82.7%) of the 295 lower limbs. This rate was higher for imaging studies which demonstrated that TVI was recognized in all examined limbs (72/72). Conversely, in cadaveric studies, the TVI was identified in 172 (77.13%) of the 223 lower limbs.

### 3.5. Anatomy

**Figure 4:**
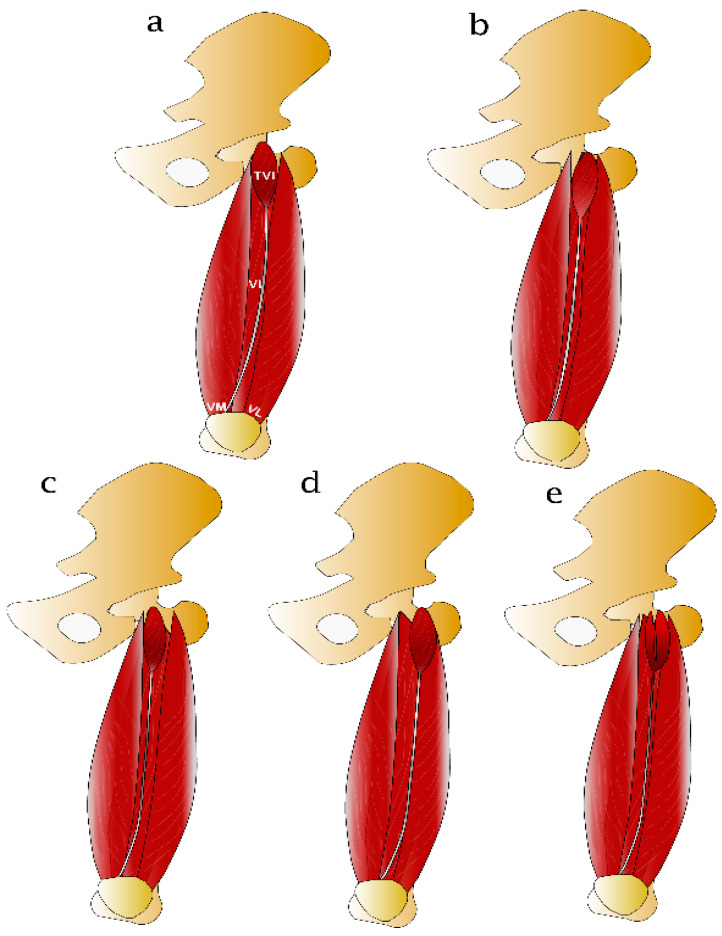
Schematic representation of the different types of the tensor of vastus intermedius and its variations with respect to interactions with the VI and VL. (**a**) Independent type, (**b**) common type, (**c**) VI type, (**d**) VL type, (**e**): multiple heads, VI: vastus intermedius, VL: vastus lateralis.

Anatomy of TVI musculature has been detailed described by five cadaveric studies [10,11,12,15,16]. The TVI was mostly identified in the anterior compartment of the thigh as a clearly separate muscle belly between VL and VI musculatures [10,12]. The morphology of the TVI presented great variability and sometimes differed in morphology between individuals and body sides [11,15]. Proximally, the TVI arises from the anteroinferior aspect of the greater trochanter of the femur distal to the intertrochanteric line and it is covered by RF [10,12]. In some cases, more complex anatomy has been reported including a similar origin with other muscles such as VL, VI and gluteus minimus (rarely) as well as an origin with multiple heads with one or more tendons [11]. Medially, TVI has a broad and flat aponeurosis that is separable, while in some cases is hardly distinguishable from VI and/or VL aponeuroses [10]. Distally, the aponeurosis forms a tendinous structure that takes an oblique course and attaches to the upper medial aspect of patella and it fuses with either the VL, VI, RF, or a combination of two or all three aponeuroses [10,12]. A schematic representation of the most frequent course of the TVI musculature in relation to the surrounding muscles is depicted in Figure 4.

### 3.6. Variations

Relative prevalence of TVI variations is summarized in Table 3. Five studies have reported morphological variations of TVI [10,11,12,15,16]. First, some studies [10,12,16] concluded that the TVI has four distinct morphological variations based on its aponeurosis course (Figure 4). Type 1 or independent type refers to the case where the aponeurosis and muscle belly are clearly distinguished from the aponeuroses of VI and VL. Type 2 (or VI type) and type 3 (or VL type) refer to the cases where the muscle is recognizable but the TVI aponeurosis is inseparable from VI and VL, respectively. Type 4 (or common type) refers to the case where the TVI belly origin is hardly recognizable while the aponeuroses is distinguished from VL and VI [10,12,16]. A recent study [15] did not verify the above classification, owning to the absence of TVI in some specimens. More recently, Olewnik et al. [11] proposed a different TVI classification based on the proximal attachment of the muscle belly [11]. Based on this scheme, three TVI variants were identified. Type I and Type II and their subtype classifications are partly similar to those described by previous studies [10,12]. Type III is mainly based on the presence of supplementary heads. Although Grob et al. [10] and Veeramani and Gnanasekaran [12] have documented the presence of multiple TVI heads, they did not take this into consideration in their final classification. Detailed descriptions of TVI variations reported by each included study can be found at Supplementary Materials.

### 3.7. Morphological Characteristics

Table 4 outlines the morphology of TVI muscle belly. Only five of the included studies have provided information for TVI muscle belly morphological characteristics such as length, width, thickness, and cross-sectional area (CSA) [11,12,16,18,20]. In particular, muscle length ranged from 101.86 ± 16.96 mm to 145.40 ± 37.55 mm [11,12,16] while the muscle width and thickness (reported in one study) was 16.76 ± 5.55 mm and 4.19 ± 1.44 mm, respectively [11]. Additionally, TVI CSA was evaluated in two separate measurement sites along the femur length [18,25]. In the proximal site, it was ranged from 1.31 ± 0.09 cm^2^ to 1.32 ± 0.16 cm^2^, while distally it ranged from 1.21 ± 0.10 cm^2^ to 1.22 ± 0.14 cm^2^ [18,25].

TVI tendon morphological characteristics were assessed by four studies [11,12,16,18]. As Table 5 illustrates, tendon length ranged from 193.55 ± 42.32 mm to 213.91 ± 34.93 mm while the muscle-tendon junction width and thickness (reported in one study) was 14.21 ± 5.92 mm and 1.70 ± 1.08 mm, respectively [11]. Tendon CSA was greater proximally (range 0.210 ± 0.018 cm^2^) than in the middle (0.199 ± 0.018 cm^2^) and distal (0.192 ± 0.016 cm^2^) locations [18].

### 3.8. Neurovascular Supply

The TVI’s vascularization and innervation were evaluated in four of the included studies mainly by simple observation of the dissected lower limbs [10,12,15] while only one study used stereomicroscopy [16]. These studies documented that TVI is vascularized independently through individual branches of the transverse branches of the lateral circumflex femoral artery and side branches of the ascending branch of the lateral circumflex femoral artery [10,12]. Additionally, it has been demonstrated that proximally TVI is innervated by individual branches which arise from the lateral side of the posterior divisions of the femoral nerve [10,12]. Ogami-Takamura et al. [16] commented that TVI innervation depends on its anatomical variation type. For instance, they found that the VL, VI and independent VL types were innervated only by nerves from the VL musculature while dual supply of nerves from the VL and VI was observed only in the common type.

### 3.9. Functional Anatomy

None of the previous studies have directly examined the function and the contribution of TVI in the lateral extensor apparatus of the knee.

## 4. Discussion

The aim of the present review was to examine and synthetize the available literature regarding the prevalence, anatomical variations and morphological characteristics of the TVI. The review showed a few high-quality experimental studies on TVI anatomy. Based on the included studies, the main findings were firstly, that the TVI is a separate muscle which is identified in most humans, secondly, it displays important interindividual variability and finally, the reviewed evidence failed to identify its functional role. To the best of our knowledge, this is the first study that systematically examined the available literature regarding this musculature.

The present review found a consistent identification of TVI in most of the included studies [10,12,16,17,18,25]. In particular, the identification of TVI as a separate anatomical structure among the other quadriceps muscles was reported in both cadaveric [10,11,12,15,16] and medical imaging studies [17,18,20,21] (Table 2). Furthermore, the appearance of an accessory muscle between the VL and VI has been documented in past anatomical studies [13,14]. Nevertheless, these investigations have not demonstrated the present of this accessory muscle universally. The inability of past anatomical studies to identify the TVI may be due to the close contact with VL and VI proximally at the thigh, along with the complex network of vessels and nerves observed at this region and as a result this musculature could be distinguished from the surrounding structures only after the neurovascular structures were traced carefully [10,24]. Furthermore, the great morphological variability displayed by the TVI as well as the absence of major surgical procedures involving the hip joint could have prevented previous investigations from recognizing the TVI as a distinct musculature among its adjacent musculatures [10,12]. Another explanation may be the lack of the detailed anatomical evaluation over the years at the proximal thigh as many anatomical dissections of the quadriceps have mostly focused on the morphological characteristics of the extensor apparatus in the distal region [5,6].

A unique characteristic of this muscle is its great morphological variability [10,11,12]. The variations of TVI musculature have been categorized in two ways, based on its aponeurosis/tendon course [10,12,16] and on its proximal attachment [11] (Supplementary Materials). This phenomenon is not uncommon, as previous investigations have revealed similar structural diversity regarding the origin of VI [26] as well as the degree of fusion between the VL and VI musculatures [27]. Furthermore, these morphological variabilities especially in TVI’s aponeurosis can be explained by the differences in the extent and location of the fusion between the aponeuroses of VL and VI with the TVI [28]. Although TVI presented a complex anatomy proximally, it is easily distinguishable in most cases at the distal aspect of the thigh [10,12]. Clinical awareness and detailed knowledge of TVI variations is necessary for medical professionals such as surgeons and radiologists, especially when performing procedures in the anterior compartment of the thigh.

If the TVI is a separate muscle head in the frontal thigh area, then the question is why it exists. Up to now, the precise functional contribution of TVI to the whole quadriceps muscle group remains unclear as none of the previous studies have directly evaluated the function and biomechanics of TVI. Nevertheless, based on its architecture, it can be suggested that the TVI is essential to counteract the forces of the medial components of the quadriceps muscle group [10,12]. Another suggestion is that the TVI seems likely to control the movement of the patella because of its course from the anterolateral aspect of the greater trochanter to the medial aspect of the patella [10,12]. Additionally, owing to the close relationship between TVI and VI aponeuroses, it has been hypothesized that it could exert tension of the VI and medialize its action [10]. Furthermore, considering its small CSA, approximately 4 to 24 times that of the other heads, it has been proposed that the contribution of TVI to the whole quadriceps muscle force generation capacity is rather limited [25]. This may explain the absence of a significant association between TVI CSA and quadriceps isometric force [29]. From the examined evidence, it appears that that this muscle is innervated and vascularized separately from the adjacent muscles [10,12,16]. This indicates that recruitment of this muscle-tendon unit which is independent from the adjacent muscles is possible. However, unless muscle activation or function when performing in vivo measurements are taken, no safe conclusions can be made about the functional importance of TVI. Techniques such as intramuscular electromyography or ultrasonography can be used for this purpose.

The results of this systematic review showed that almost half of the included studies were ranked as having “High” risk of bias according to the AQUA checklist. For example, several studies did not provide precise descriptions of and measurements of TVI morphology while subject or specimen characteristics especially in cadaveric studies were poorly described. This indicates that the findings reported by the included studies should be considered with some caution. Furthermore, the amount of evidence is relatively small as it is based on approximately 300 limbs. Given the variability in TVI morphology a larger number of samples is required to reach generalized conclusions regarding the morphology of TVI and its variants. Additionally, future work could use advanced imaging techniques with larger sample sizes, including both healthy and individuals with lower limb injuries, to gain a clear understanding of TVI functional properties. Greater demographic data would enable the assessment of gender- or side-based differences of the examined population. Owing to the paucity of information concerning the embryology of TVI, it is important that further studies should evaluate whether this musculature has a specific embryological origin. Finally, future studies could provide information about the distal attachment of TVI as different insertion levels at the patella could influence TVI function.5.

## 5. Conclusions

This systematic review illustrated that there are a few high-quality studies that examined TVI anatomy and morphological characteristics. These studies showed that the TVI was identified in the vast majority of the examined limbs with a defined origin and insertion even though in some cases they presented more variable connections with adjacent structures. Furthermore, TVI muscle architecture presented great complexity that varied along its length. Therefore, future investigations are suggested to gain a clearer understanding of this musculature.

## Figures and Tables

**Table 1:** Summary of the papers included in this systematic review.

Study	Bonnechère et al. [15]	Grob et al. [10]		Grob et al. [24]	Ogami-Takamura et al. [16]
Subject/Specimen characteristics	20 limbs, 4 M and 6F Age N/R	26 limbs, 9 M and 7 F, Age N/R		1 lower limb	35 lower limbs, 19 M and 16 F, Age 61–98 years
Pathology	N/R	N/R		N/R	Without any injury
Dissection technique	From the supine position, three skin incisions were performed: from the anterior superior iliac spine to the pubic tubercle, a horizontal incision below the tibial tuberosity and a vertical incision between these two incisions. Different muscle bellies were identified manually, and different parts were noted only if it was possible to separate the different heads without scalpel. *	From supine position, the anterior aspect of the hip joint was approached. Primarily, the ascending branch of the LCFA was recognized and traced medially. TVI identification was traced from their origin to their insertion. *		Dissection of all lower limb muscles. Muscle bellies of the extensor apparatus were traced from their origins to their insertion into the quadriceps tendon, and their affiliation was assessed. *	Line connecting the center of the inguinal ligament to the patella was incised. VL, VM and VI the TVI were described. *
Imaging technique	N/R	N/R		MRI	15 lower limbs, using computed tomography
Measurement recorded	Lateral extensor apparatus with emphasis on TVI identification, origin, insertion and innervation.	Observation and tracing of neurovascular supply of TVI to determine its relationship with the VL and VI. Examination of TVI anatomy with respect to its location, origin and insertion. No quantification of TVI dimension was reported.		Description οf lateral knee extensor apparatus and TVI with focus on conjunction between the TVI, VI, and VL. No quantification of TVI dimensions.	TVI belly and aponeurosis length using a vernier caliper. Descriptions of TVI location, origin, insertion and innervation
Investigators	N/R	N/R		N/R	N/R
Subject/Specimen characteristics	106 lower limbs 34 M and 19 F, Mean Age 68.4 years	20 subjects (40 lower limbs), 10 M and 10 F	21 subjects, (21 lower limbs), 17 M and 4 F, Mean Age 21.72 ± 1.83 years	10 subjects (10 lower limbs), 10 M and F, Mean age 21.6 ± 2.41 years	36 lower limbs, 27 M and 9 F
Pathology	Any lower limbs with evidence of surgical intervention in the dissected area were excluded	N/R	No lower limb injury	No lower limb injury	No lower limb injury
Dissection technique	From the supine position, the hip joint capsule was resected and the inguinal ligament identified. Muscle belly’s origin and insertion were identified. *	N/R	N/R	N/R	A longitudinal incision from mid-inguinal point to the patella. Muscle components were defined by blunt dissection. *
Imaging technique	N/R	Ultrasound	Ultrasound	Extended field of view ultrasound	N/R
Measurement recorded	Length, width, thickness of belly and muscle-tendon unit junction using an electronic digital caliber.	Descriptions of TVI belly and tendon relationship with adjacent structures. Dimensions not reported.	CSA along TVI belly and tendon	TVI CSA at two sites	TVI belly and aponeurosis length and distance of fusion of TVI from the patella to VL or VI using a digital vernier caliper. Description of TVI location, origin, insertion and neurovascular supply
Investigators	N/R	N/R	Experienced investigator	Experienced investigator	N/R

M: Males, F: Females, LCFA: lateral circumflex femoral artery, QF: quadriceps femoris, TVI: tensor of vastus intermedius, MRI: magnetic resonance images, VL: vastus lateralis, VI: vastus intermedius, VM: vastus medialis, CSA: cross-sectional area, N/R: Not Reported, * In all dissection studies, TVI was exposed after cutting and lifting fascia, sartorius and RF as well as femoral nerve branches and vessels.

**Table 2:** Prevalence of tensor of the vastus intermedius between the studies.

Study	TVI/n Lower Limbs	% Prevalence of TVI
Cadaveric studies		
Bonnechère et al. [15]	7/20	35
Grob et al. [10]	26/26	100
Ogami-Takamura et al. [16]	35/35	100
Olewnik et al. [11]	68/106	64.1
Veeramani and Gnanasekaran [12]	36/36	100
Imaging Studies		
Grob et al. [24]	1/1	100
Rajasekaran and Hall [17]	40/40	100
Sahinis et al. [18]	21/21	100
Sahinis et al. [25]	10/10	100
Total	244/295	82.7

TVI: tensor of vastus intermedius.

**Table 3:** Prevalence of tensor of vastus intermedius variations between anatomical studies.

Studies	n Lower Limbs that TVI Identified	Independent Type (%)	VI Type (%)	VL Type (%)	GM Type (%)	Common Type (%)	Multiple Heads of TVI
Bonnechère et al. [15]	7	14	-	86	-	-	-
Grob et al. [10]	26	42.31	23.08	19.23	-	15.38	5 cases
Ogami-Takamura et al. [16]	35	11	23	37	-	29	-
Olewnik et al. [11] *	68	44.1	4.5	23.5	2.9	-	25%
Veeramani and Gnanasekaran [12]	36	33.33	8.33	30.56	-	27.78	3 cases

TVI: tensor of vastus intermedius, VL: vastus lateralis, VI: vastus intermedius, GM: gluteus minimus, * = the multiple heads of TVI included at the final classification of its anatomical variations.

**Table 4:** Results of architectural data for the muscle belly of tensor of the vastus intermedius.

				Muscle Cross-Sectional Area (cm^2^)	
Study	Muscle Length (mm)	Muscle Width (mm)	Muscle Thickness (mm)	Proximal Site	Distal Site
Ogami-Takamura et al. [16]	102.36 ± 34.05	-	-	-	-
Olewnik et al. [11]	101.863 ± 16.96	16.76 ± 5.55	4.19 ± 1.44	-	-
Sahinis et al. [18]	-	-	-	1.32 ± 0.16	1.22 ± 0.14
Sahinis et al. [25]				1.31 ± 0.09	1.21 ± 0.10
Veeramani and Gnanasekaran [12]	145.40 ± 37.55	-	-	-	-

**Table 5:** Results of architectural data for the tendon/aponeurosis of tensor of vastus intermedius.

		Muscle-Tendon Junction		Tendon Cross-Sectional Area (cm^2^)		
Study	Tendon Length (mm)	Tendon Width (mm)	Tendon Thickness (mm)	Proximal Site	Middle Site	Distal Site
Ogami-Takamura et al. [16]	208.50 ± 36.03	-	-	-	-	-
Olewnik et al. [11]	213.91 ± 34.93	14.21 ± 5.92	1.70 ± 1.08	-	-	-
Sahinis et al. [18]	-	-	-	0.21 ± 0.01	0.19 ± 0.01	0.19 ± 0.01
Veeramani and Gnanasekaran [12]	193.55 ± 42.32	-	-	-	-	-

## Data Availability

The authors confirm that the data supporting the findings of this study are available upon request.

## Supplementary Materials

The following are available online at https://www.mdpi.com/article/10.3390/jfmk6030077/s1, Table S1: Morphological variations classification of Tensor of vastus intermedius based on its tendon/aponeurosis course.
