# Supplementary material for: Anatomy, Morphology and Function of the Tensor of Vastus Intermedius: A Systematic Review
Source: J Funct Morphol Kinesiol. 2021 Sep 16;6(3):77. doi: 10.3390/jfmk6030077 (PMC8482252; doi:10.3390/jfmk6030077)
Supplement: Supplementary file 1 [file jfmk-06-00077-s001.zip › jfmk-1356316-supplementary.pdf]

Table S1. Morphological variations classification of Tensor of vastus intermedius based on its tendon/aponeurosis course.

	Type I	Type II	Type III	Type IV
**Based on aponeurosis/tendon course**				
Proximal (Origin)	From the upper part of the intertrochanteric line and anterior part of greater trochanter of femur, separable from VL origin. (Independent type)	From the anterior and lateral surfaces of the upper two-thirds of the femoral shaft together with VI. The anterior border is distinguishable while the posterior border is fused with VI. (VI type)	From the upper part of the intertrochanteric line and anterior part of greater trochanter together with VL. (VL type)	Is not clearly recognizable from both VL and VI as they have common origin. (Common type)
Middle (Aponeurosis)	Separable from both VI and VL.	Separable from VL but not from VI.	Separable from VI but not from VL.	Separable from both VI and VL.
Distal (Attachment)	Common for all the types at the upper medial aspect of patella fusing with either the VL, VI, RF, or a combination of two or all the three aponeuroses.			
**Based on the proximal attachment of the muscle belly**				
	Type I	Type II	Type III	
Proximal (Origin)	From the upper part of the anterior surface of greater trochanter, the intertrochanteric and gluteus medius ridge where the muscle belly runs laterally in relation to the VI for subtype IA and medially for subtype IB (Independent type)	IIA: from VL<br>IIB: from VI<br>IIC: from gluteus minimus	IIIA: two heads (lateral and medial) with a single common tendon.<br>Lateral head: from the upper part of the anterior surface of greater trochanter, the intertrochanteric and gluteus medius ridge.<br>Medial head: from the anterior surface of the thigh above the VI proximal attachment.<br>IIIB: two heads (lateral and medial) with two distinct tendons.<br>Lateral head: similar origin as IIIA<br>Medial head: from the anteromedial surface of the thigh above the VI proximal attachment.<br>IIIC: three heads (lateral, intermediate and medial)<br>Lateral and intermediate: from the VL<br>Medial head: similar origin as lateral head of type IIIA and IIIB<br>IIID: four heads (bifurcated lateral and bifurcated medial).<br>Lateral bifurcated: from the lower part of the anterior surface of greater trochanter, intermediate part of the VL and from the antero-lateral sur-face of the shaft of the femur<br>Medial bifurcated: from the innominate tubercle of the femur and the lower part of the anterior surface of greater trochanter	
Middle (Aponeurosis)	Courses medially for both subtypes	-	-	-
Distal (Attachment)	-	-	-	-

TVI: Tensor of Vastus Intermedius, VI: Vastus Intermedius, VL: Vastus Lateralis, RF: Rectus Femoris,
